# Idiopathic chronic fatigue in older adults is linked to impaired mitochondrial content and biogenesis signaling in skeletal muscle

**DOI:** 10.18632/oncotarget.10685

**Published:** 2016-07-18

**Authors:** Nicholas R. Wawrzyniak, Anna-Maria Joseph, David G. Levin, David M. Gundermann, Christiaan Leeuwenburgh, Bhanuprasad Sandesara, Todd M. Manini, Peter J. Adhihetty

**Affiliations:** ^1^ Department of Applied Physiology and Kinesiology, University of Florida, Gainesville, Florida, USA; ^2^ Department of Aging and Geriatric Research, Division of Biology of Aging, University of Florida, Gainesville, Florida, USA

**Keywords:** fatigue, skeletal muscle, mitochondria, PGC-1α, Gerotarget

## Abstract

Fatigue is a symptom of many diseases, but it can also manifest as a unique medical condition, such as idiopathic chronic fatigue (ICF). While the prevalence of ICF increases with age, mitochondrial content and function decline with age, which may contribute to ICF. The purpose of this study was to determine whether skeletal muscle mitochondrial dysregulation and oxidative stress is linked to ICF in older adults. Sedentary, old adults (*n* = 48, age 72.4 ± 5.3 years) were categorized into ICF and non-fatigued (NF) groups based on the FACIT-Fatigue questionnaire. ICF individuals had a FACIT score one standard deviation below the mean for non-anemic adults > 65 years and were excluded according to CDC diagnostic criteria for ICF. Vastus lateralis muscle biopsies were analyzed, showing reductions in mitochondrial content and suppression of mitochondrial regulatory proteins Sirt3, PGC-1α, NRF-1, and cytochrome c in ICF compared to NF. Additionally, mitochondrial morphology proteins, antioxidant enzymes, and lipid peroxidation were unchanged in ICF individuals. Our data suggests older adults with ICF have reduced skeletal muscle mitochondrial content and biogenesis signaling that cannot be accounted for by increased oxidative damage.

## INTRODUCTION

“As a self-reported measure, fatigue is complex and multidimensional and, not surprisingly, has been defined in a number of ways: as a feeling that interferes with usual functioning and has a multifactorial origin, as a sense of diminished energy and increased need to rest, and as physical or mental weariness resulting from exertion. As such, definitions of fatigue frequently vary across studies, across diseases, and even between investigators and patients,” [1].

Systemic fatigue (here on referred to as “fatigue”) typically manifests itself in association with a wide variety of clinical pathologies including cancer, congestive heart failure, sleep disorders, mental disorders, and fibromyalgia [1]. Chronic fatigue presents as an overall lack of systemic energy that persists for numerous months and cannot be alleviated by prolonged rest, leading to functional declines in daily living activities [1-3]. Additionally, the clinical definition of chronic fatigue syndrome (CFS) must exhibit at least four of the following symptoms to qualify as CFS, which include: impairment of short-term memory or concentration, sore throat, tender lymph nodes, muscular pain, joint pain, headaches, unrefreshing sleep, and post-exertional malaise. However, there are individuals who report or exhibit sustained fatigue in the complete absence of any underlying medical conditions and this is referred to as “idiopathic chronic fatigue” (ICF), which is different from CFS. ICF is defined as clinically diagnosed psychological and physiological fatigue with no known underlying pathologies and does not need to meet four of the six criteria of CFS [2]. Although no known etiology exists for ICF, it has been speculated to stem from metabolic abnormalities within various tissues, particularly skeletal muscle, but the molecular details involved have yet to be elucidated [1, 4, 5]. These tissues decline as part of the “aging process,” while the incidence of ICF is higher in elderly individuals [1], suggesting there may be age-related metabolic changes that could exacerbate symptoms of fatigue.

Abnormalities in mitochondrial regulation and function in muscle tissue are potential areas worth exploring for a causal relation to ICF since this organelle plays an integral role in regulating bioenergetics and oxidative capacity, particularly in high energy-demanding tissues [6]. Mitochondrial content is maintained *via* a process termed mitochondrial biogenesis and involves the coordination of the nuclear and mitochondrial genomes. This is orchestrated by the transcriptional coactivator peroxisome proliferator-activated receptor gamma coactivator-1 alpha (PGC-1α) that acts to increase the expression of numerous genes involved in mitochondrial biogenesis including nuclear respiratory factors 1 and 2 (NRF-1, NRF-2) and mitochondrial transcription factor A (TFAM) [7-11]. Additionally, mitochondrial morphology proteins of fission (Drp-1, Fis-1) and fusion (Mfn-1, Mfn-2) are critical for maintaining cycles of mitochondrial turnover, and recycling of mitochondrial constituents [12-15]. Mitochondrial biogenesis is also associated with nascent formation of electron transport chain (ETC) complexes, which are crucial for the production of ATP (energy) *via* cellular respiration. Thus, dysregulation of ETC complexes may be linked to ICF because chronic fatigue is often described as a “lack of energy”. Impairments in mitochondrial biogenesis, regulatory processes and overall mitochondrial function are involved in the pathophysiology of diabetes, Alzheimer's, Parkinson's, Huntington's disease, sarcopenia, and normal aging [16-22]. However, it is currently unknown whether impairments in mitochondrial biogenesis and/or mitochondrial quality control pathways contribute to the etiology of ICF in older adults.

Aging without overt disease is associated with significant impairments in the size, function, and metabolic profile of skeletal muscle [20, 23-25]. Although there are a variety of underlying mechanisms contributing to age-related skeletal muscle dysfunction, aberrations in mitochondrial function and regulation appears to be a central factor because of the mitochondrion's role in cellular energy production (i.e. ATP), origin of damaging free radicals, impaired autophagy and mitochondrially-mediated apoptosis [19, 26-30]. Currently, only a few studies have investigated whether mitochondrial abnormalities contribute towards dysregulated muscle bioenergetics in younger patients with chronic fatigue. Smits et al. (2011) showed that skeletal muscle from younger patients with chronic fatigue syndrome (CFS) exhibited no change in mitochondrial respiratory chain complex activities or ATP production but overall mitochondrial content was significantly reduced compared to controls [31]. Additionally, a study investigating middle-aged patients diagnosed with CFS found reduced mitochondrial function, higher levels of lipid peroxidation, but similar numbers of mitochondria in white blood cells when compared to healthy controls [32]. Furthermore, patients with CFS have elevated markers of oxidative damage in muscle [33] and plasma [34], which may stem from the overproduction of damaging free radicals resulting from impaired mitochondrial function. Despite this limited evidence for mitochondrial involvement in CFS, there are no studies to date that have comprehensively investigated mitochondrial function and regulation in an aged population with ICF [35].

In the present study, we tested the hypothesis that older adults presenting with ICF would exhibit lower mitochondrial content, function, and impaired regulation in skeletal muscle when compared to NF counterparts. Specifically, we hypothesized that older adults presenting with ICF would exhibit lower skeletal muscle mitochondrial enzymatic activity, number, gene expression, bioenergetic regulation and quality control (fission & fusion) compared to age-matched NF older adults. Importantly, our study used stringent eligibility criteria, analogous to the criteria for diagnosing CFS such that outright and subclinical comorbidities commonly associated with fatigue were excluded from the study to minimize the contamination of underlying medical conditions.

## RESULTS

### Participant characteristics

The main participant characteristics are provided in Table 1. A total of 48 individuals (age 72.4 ± 5.3 years) participated in the study that included 20 idiopathic chronically fatigued (ICF) individuals and 28 non-fatigued (NF) age-matched counterparts. Most participants were Caucasian (95%) and consisted of a similar number of males (*n* = 26) and females (*n* = 22). As expected, fatigued participants had higher FACIT-F scores, but there were no differences in age, gender, and ethnicity were observed between the two groups. The ICF group had a slightly higher (12%) body mass index, reported more depression-like symptoms on the CES-D and was more likely to be treated for hypertension. Regarding blood counts, the ICF group had higher levels of neutrophils and lower aspartate transaminases, lymphocytes, basophils and red cell distribution.

**Table 1:** Descriptive participant characteristics

Characteristics mean (SD) or No. (%)	NF (n = 28)	ICF (*n* = 20)	*p*-value
**Age (yrs.)**	72.4 (4.9)	72.4 (5.9)	0.99
**Caucasian**	26 (92.9)	20 (100.0)	0.48
**Female**	13 (48.2)	9 (45.0)	0.83
**College education**	22 (78.6)	17 (85.0)	0.57
**Live alone**	10 (35.7)	5 (25.0)	0.43
**MMSE score (0-30)**	28.4 (1.7)	28.2 (1.9)	0.71
**Body Mass Index (kg/m^2^)**	25.5 (4.1)	27.7 (2.6) *	0.04
**FACIT-F score (0-50)**	49.5 (2.5)	24.3 (6.1) *	<0.01
**Excellent/very good health**	26 (92.9)	13 (65.0) *	0.02
**Unable to walk one mile**	1 (3.6)	2 (10.0)	0.36
**CES-D score (0-60)**	3.68 (3.9)	10.1 (4.4) *	<0.01
**History of cardiovascular disease◆**	4 (14.3)	3 (15.0)	0.95
**Osteoarthritis**	2 (7.14)	4 (20.00)	0.18
**History of cancer**	7 (25.0)	7 (35.0)	0.45
**Hypertension**	11 (40.7)	14 (70.0) *	0.05
**Diabetes**	3 (10.7)	5 (25.0)	0.19
**Fasting glucose (mg/dL)**	95.6 (12.8)	106.6 (21.7) *	0.04
**Albumin (g/dL)**	4.4 (0.2)	4.5 (0.3)	0.07
**Aspartate transaminase (IU/L)**	25.8 (6.6)	22.1 (3.0)	0.02
**Alanine transaminase (IU/L)**	24.4 (9.3)	21.2 (5.8)	0.18
**Platelets**	252.4 (52.2)	243.6 (60.6)	0.59
**White blood cell count**	5.8 (1.6)	6.0 (0.8)	0.64
**Neutrophils**	56.3 (7.7)	61.8 (5.6)	0.01
**Lymphocytes**	30.0 (7.4)	26.1 (4.8) *	0.04
**Monocytes**	6.6 (1.6)	6.5 (1.5)	0.83
**Eosinophils (%)**	3.7 (2.0)	3.0 (2.0)	0.27
**Basophils (%)**	0.9 (0.5)	0.6 (0.3)	0.02
**Hemoglobin (g/dL)**	14.3 (0.9)	14.2 (1.3)	0.80
**Hematocrit**	42.8 (2.7)	42.7 (3.7)	0.96
**Red blood cells**	4.6 (0.4)	4.6 (0.3)	0.75
**Corpuscular volume**	93.5 (5.8)	92.3 (4.6)	0.45
**Corpuscular hemoglobin**	31.2 (1.8)	30.6 (1.6)	0.28
**Red cell distribution**	12.5 (0.7)	13.0 (0.7) *	0.04

MMSE = Mini-Mental State Exam. FACIT-F = Functional Assessment of Chronic Illness Therapy Fatigue scale. CES-D = Center for Epidemiologic Studies Depression scale. ◆CVD disease defined as a history of heart attack, coronary/myocardial infarction, stroke/brain hemorrhage, heart failure/congestive heart failure or abnormal heart rhythm. Values are reported as means ± SD for continuous variables and or n (%) for categorical variables. Significance set at *P < 0.05.

### Skeletal muscle mitochondrial content

**Figure 1:**
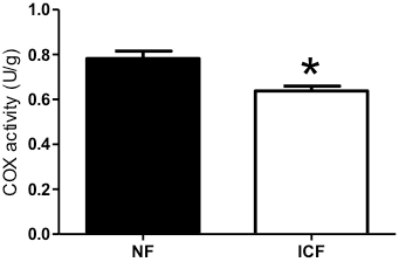
Mitochondrial content Mitochondrial content was measured by Cytochrome *c* oxidase (COX) activity (*n* = 17-26 per group) from vastus lateralis muscle biopsies of ICF and NF individuals. Values are means ± SEM with significance set at *P < 0.05.

Reductions in mitochondrial content and bioenergetics are a common feature of skeletal muscle dysfunction in a number of conditions including but not limited to aging, cachexia, and sarcopenia. Cytochrome *c* oxidase (COX) activity, a common marker of mitochondrial content [36] was significantly lower (18.4%) in ICF individuals compared to NF (Figure 1).

### Mitochondrial biogenesis signaling

**Figure 2:**
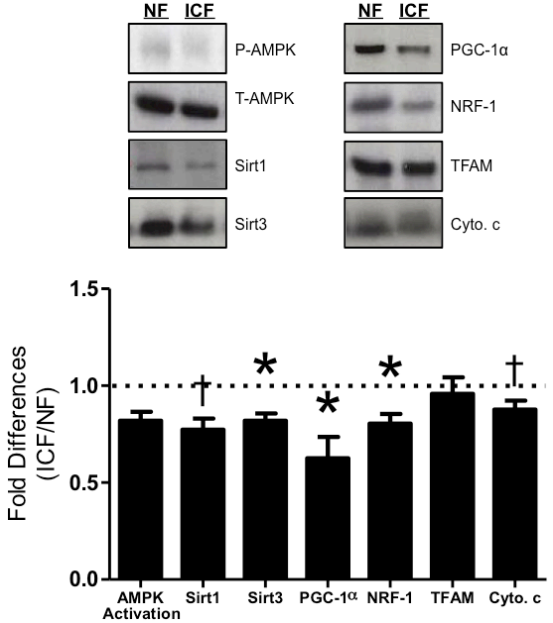
Mitochondrial biogenesis signaling Western blot analysis of key biogenesis signaling proteins including AMPK = 5' adenosine monophosphate-activated protein kinase; Sirt1 = sirtuin 1; Sirt3 = sirtuin 3; PGC-1α = peroxisome proliferator-activated receptor gamma coactivator-1 alpha; NRF-1 = nuclear respiratory factor 1; TFAM = mitochondrial transcription factor A; Cyto. c = cytochrome c. AMPK activation was determined as ratio of phosphorylated protein (p-AMPK) compared to total protein (t-AMPK). Graphical summary of repeated experiments is shown below. Values are represented as means ± SEM; *n* = 16-28 per group. *P < 0.05; †*P* < 0.10. Data are expressed as fold difference (ICF/NF).

AMPK (5' adenosine monophosphate-activated protein kinase) activation, measured by phosphorylated-AMPK over total-AMPK, was not altered in ICF individuals compared to the NF group (Figure 2). Levels of the mitochondrially-localized Sirt3 protein were lower (18.1%; *P* < 0.05) in ICF individuals compared to NF, while there was a trend for lower levels of Sirt1 protein (*P* = 0.08; Figure 2). PGC-1α expression was also significantly lower (37.4%; *P* < 0.05) in ICF individuals (Figure 2), as were levels of its downstream target, NRF-1 (19.4%; *P* < 0.05; Figure 2). No differences were observed in TFAM, but there was a trend for lower expression of cytochrome c in the ICF group (*P* = 0.09; Figure 2).

### Electron transport chain complexes

**Figure 3:**
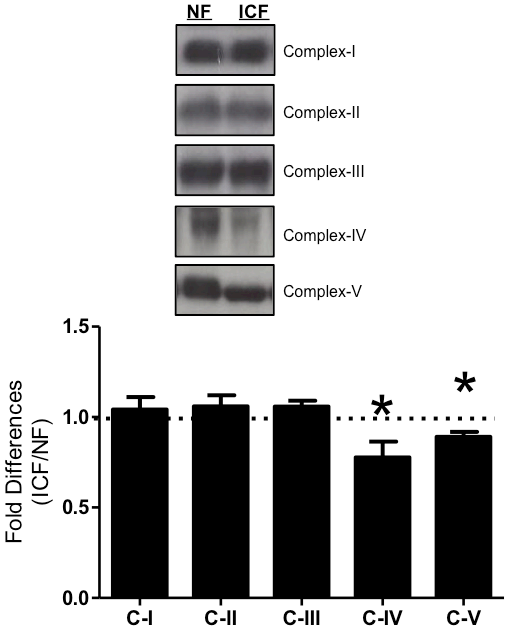
ETC complexes Expression levels from subunit proteins of ETC complexes I-V were measured *via* western blot. These included CI - subunit NDUFB8 (nuclear encoded), CII - 30kDa (nuclear), CIII - core protein 2 (nuclear); CIV - subunit I (mtDNA encoded); CV - alpha subunit (nuclear). Summary of repeated experiments is shown below. Values are represented as means ± SEM; *n =* 18-28 per group. Significance set at *P < 0.05. Data are expressed as fold difference (ICF/NF).

Levels of ETC complexes I-III were not altered in ICF individuals compared to NF counterparts. However, Complex IV (cytochrome c oxidase) and Complex V (ATP synthase) were both significantly lower (22.3%, *P* < 0.05; 9.9% *P* < 0.05) in ICF patients (Figure 3).

### Mitochondrial morphology proteins

**Figure 4:**
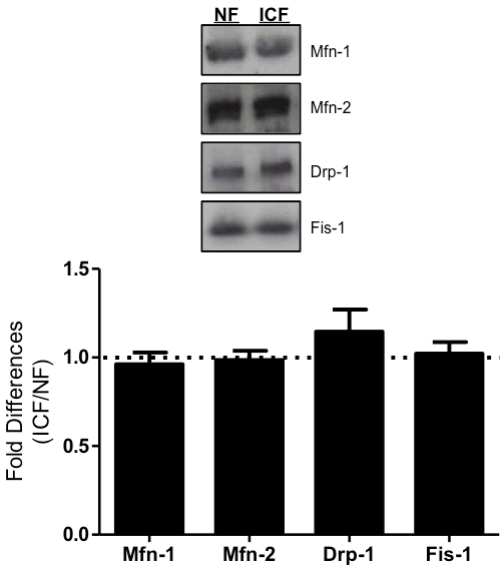
Mitochondrial morphology Mitochondrial fusion (Mfn-1 = mitofusin 1; Mfn-2 = mitofusin 2) and fission proteins (Drp-1 = dynamin-related protein 1; Fis-1 = fission protein 1) were measured via western blot. Graphical summary of repeated experiments is shown below. Values are represented as means ± SEM; *n* = 15-27 per group. Significance set at *P < 0.05. Data are expressed as fold difference (ICF/NF).

ICF and NF individuals had similar levels of mitochondrial fusion proteins mitofusin 1 (Mfn-1) and mitofusin 2 (Mfn-2), and mitochondrial fission proteins dynamin-related protein 1 (Drp-1) and fission protein 1 (Fis-1) (Figure 4).

### Oxidative damage and antioxidant capacity

**Figure 5:**
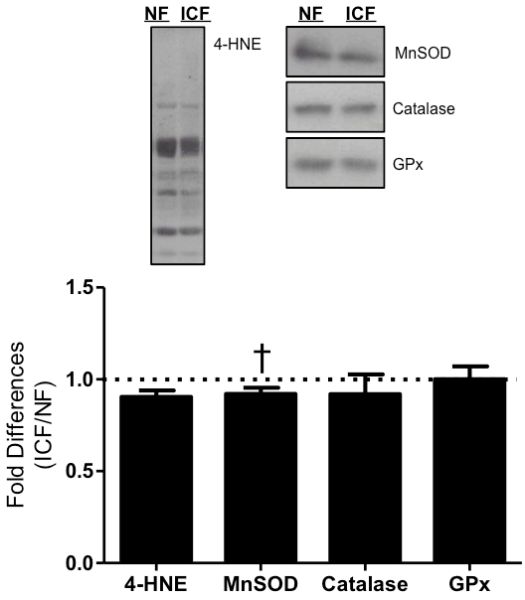
Oxidative damage and antioxidants Lipid peroxidation (4-HNE = 4-hydroxynonenal) and expression of critical antioxidant enzymes (MnSOD = Manganese superoxide dismutase; Cat = catalase; and GPx = glutathione peroxidase) were measured *via* western blot. Summary of repeated experiments are depicted below. Values are represented as means ± SEM; *n* = 18-27 per group. †*P* < 0.10. Data are expressed as fold difference (ICF/NF).

Lipid peroxidation, measured as 4-hydroxynonenol (4-HNE), was similar between the ICF and NF groups (Figure 5). Although manganese superoxide dismutase (MnSOD) tended to be lower in ICF individuals (9.9%, *P* = 0.07), there were no statistically significant differences to support antioxidant species catalase (Cat) and glutathione peroxidase (GPx) as a major factor explaining fatigue in this sample population (Figure 5).

## DISCUSSION

There is limited mechanistic data to explain the presentation of ICF in older adults [4, 5]. However, mitochondrial dysfunction represents a plausible underlying mechanism for ICF since impaired function of these energy producing organelles in highly metabolic tissues, such as muscle, is associated with numerous disease states and conditions, including aging. We hypothesized that older adults presenting with ICF would exhibit lower skeletal muscle mitochondrial enzymatic activity, number, gene expression, bioenergetic regulation and quality control (fission & fusion) compared to age-matched NF older adults. To our knowledge, this is the first study to provide a detailed mechanistic analysis of mitochondrial content and signaling in skeletal muscle of older adults with ICF. The results suggest that older adults with ICF exhibit greater mitochondrial dysregulation that may reduce the muscle's ability to produce ATP and that this may contribute towards the etiology and symptoms associated with ICF.

Numerous mitochondrially-mediated mechanisms are involved with age-associated reductions in energy homeostasis, redox status, mitophagy, mitochondrial dynamics and apoptosis [17, 18, 20, 37-42]. In this study, we found older adults with ICF had reduced overall mitochondrial content in muscle compared to their age-matched NF counterparts. The explanations for these findings could be diverse, and one of which may be related to exercise and overall daily physical activity that is clearly associated with overall mitochondrial content and mitochondrial-mediated adaptations [6, 43-47]. However, both groups of participants were considered sedentary after being screened for engaging in less than 20 minutes of structured physical activity per week. Additionally, significant results were unchanged after adjusting for the variability in light, moderate and vigorous physical activity in a subset of 28 participants who wore hip accelerometers (data not shown). Despite equally sedentary behaviors, ICF individuals presented with slightly higher (but statistically significant) BMI levels than their NF counterparts. Individuals with Class II Obesity (BMI > 35) were previously shown to have reductions in skeletal muscle mitochondrial content, specifically cytochrome c oxidase levels (-32%), compared to lean (BMI < 25) age-matched counterparts [48]. Thus, slight elevations in BMI of ICF individuals may contribute to a cyclical pattern of increasingly sedentary behaviors, reduced mitochondrial content, and increased sensations of fatigue. Examination of correlations with each mitochondrial marker to BMI revealed that only TFAM was significantly correlated to BMI (*r* = 0.34, *p* = 0.02), while all others were lower than *r* = 0.15 (*p* > 0.30). Multiple regression models showed that differences between groups in mitochondrial markers remained unchanged when adjusting for BMI (data not shown). Lastly, we did observe slightly different immune profiles (basophils, lymphocytes, and neutrophils) between the two groups. However, it should be noted that these levels are well within normal ranges and would not typically be a medical concern. For example, the normal range for neutrophils is 35 - 80%. The ICF group had a mean value of 61.8%, which is well within normal ranges. This was also true for lymphocytes and basophils. Therefore, we don't believe these differences are a cause of fatigue symptoms, but may be related to anxiety caused by fatigue symptoms, which is commonly noted in older adults with depression [49, 50]. In all, our data indicate the magnitude of mitochondrial dysregulation is sufficient to detect differences between fatigue states, which are noteworthy considering that fatigue, when measured as a general state *via* a questionnaire, is complex and multidimensional.

Mitochondrial content in skeletal muscle is largely dictated by mitochondrial biogenesis processes controlled primarily *via* PGC-1α [6, 9, 10, 51, 52]. Aged muscle is associated with reductions in PGC-1α expression levels [53], mitochondrial content, and mitochondrial function [17, 37, 46, 54]. Transgenic overexpression of PGC-1α in skeletal muscle yields reduced dysfunction and suppressed cachexia with age, leading to improved strength and endurance capacity [45, 55]. In our study, we found significant reductions in PGC-1α protein levels and in its upstream activator (Sirt1) and downstream effectors (NRF-1, Sirt3) in older adults with ICF. Because Sirt3 is dependent upon the expression of PGC-1α to orchestrate antioxidant expression and mediate cellular respiratory status, such reductions can ultimately lead to disruption of cellular energy homeostasis and oxidative capacity [56]. Furthermore, activation of skeletal muscle PGC-1α was recently shown to reduce depressive symptoms, of which there is overlap with ICF symptoms, in rodents by blocking the kynurenine pathway of tryptophan degradation. PGC-1α exerts these effects by activating kynurenine acetyltransferases to convert kynurenine (a stress-induced metabolite able to cross the blood-brain barrier and contribute towards depression) into kynurenic acid (which is unable to cross the blood-brain barrier) and thus minimizing the manifestations of depressive symptoms in the brain [57]. In the present study, participants in the ICF group reported more depressive symptoms and thus had higher CES-D scores, but there were significant overlaps in the wording of the questions on the FACIT-F and CES-D questionnaires. This overlap made it difficult to delineate the differences between depression-like and fatigue-like symptoms. However, in accordance with our exclusion criteria, none of our participants were clinically diagnosed with depression. Interestingly, Kato, et al., reported, “elevated premorbid stress is a significant risk factor for chronic fatigue-like illness [in the general population],” [58]. Taken together, these data suggest that older adults with ICF have lower skeletal muscle PGC-1α, which may relate to increased fatigue symptoms through recently discovered pathways that connect muscle biomarkers to perceived fatigue symptoms in the brain.

Components of the ETC are integral to the mitochondrion's ability to maintain energy homeostasis. A vast majority of ETC proteins encoded by the mitochondrial genome are regulated by the important mitochondrial transcription factor, TFAM, which is controlled by PGC-1α *via* activation of NRF-1. Despite significant decrements in PGC-1α and NRF-1, TFAM expression levels were not altered and this coincided with unchanging expression of proteins representing complexes I-III in ICF compared to NF. However, marked decreases in the expression of complexes IV and V of the ETC were exhibited with ICF. Interestingly, these two complexes have a greater proportion of their proteins encoded by the nuclear genome compared to complexes I-III and reductions in these two complexes have significant impact on mitochondrial oxidative capacity and energy production [54]. These reductions in mitochondrial signaling proteins were relatively homogenously correlated to the 13 individual FACIT-F questions (data not shown), indicating that the summed FACIT-F score was representative of the questions individually. Collectively, our data indicate significant decreases in upstream mitochondrial signaling (i.e. PGC-1α) leads to deleterious alterations of downstream ETC complexes, which may impair the ATP producing capacity of the mitochondria because these complexes play a central role in chemiosmotic coupling [19, 59-61].

Mitochondria are a significant source of cellular reactive oxygen species (ROS) production which beyond a certain cellular threshold can lead to oxidative stress for the cell and cause damage to intracellular macromolecules such as proteins, lipids and nucleic acids. Oxidative stress and other cellular perturbations can evoke processes of fission and fusion to alter mitochondrial ultrastructure in order to preserve the viability of the organelle [12, 15, 19, 22, 62-64]. Mitochondrial morphology proteins were not different between the ICF and NF groups despite reductions in mitochondrial biogenesis signaling molecules, a finding that is corroborated by previous data from studies in PGC-1α knockout animals [36]. This suggests that mitochondrial morphology is not a process that is altered with ICF, but electron microscopy of mitochondria would be required to confirm this finding. To investigate whether oxidative damage and/or oxidative stress may contribute to ICF, we analyzed lipid peroxidation measured as 4-hydroxynonenol (4-HNE) and the expression of the antioxidant enzymes manganese superoxide dismutase (MnSOD), catalase, and glutathione peroxidase. Our data only showed a modest decrement in MnSOD with all other markers indicating no significant differences in oxidative damage or antioxidant capacity with ICF. Taken together, these findings suggest that neither oxidative damage nor alterations in mitochondrial morphology and ultrastructure appear to contribute to mitochondrial abnormalities with ICF. Thus, despite lower mitochondrial number, biogenesis signaling, and ETC enzymatic content with ICF, these impairments did not transfer to elevated oxidative damage and/or oxidative stress in muscle.

We have shown that reductions in overall mitochondrial content associated with impaired mitochondrial regulatory pathways appear to contribute towards ICF in older adults. Specifically, significant declines in the expression of upstream and downstream proteins associated with PGC-1α, as well as reduced PGC-1α itself, may be an important factor in the lack of systemic energy in these subjects. This study was limited by our analyses and interpretations on a relatively small amount of muscle biopsy tissue available to perform more comprehensive biochemical assays. Further studies investigating more detailed cellular and molecular mechanisms associated with ICF in older adults are warranted. Another limitation of this and previous epidemiological studies on ICF and aging is the possibility that self-reported fatigue in these studies are confounded by other subclinical conditions [35, 65, 66], which we attempted to rectify by employing stringent exclusion criteria and blood work in line with the CDC case definition [2]. However, subclinical diseases not captured during the screening process may continue to contribute to ICF symptoms. Although poor nutrition could be a potential cause of fatigue, we did not measure dietary intake. However, it is likely to have a low effect because there was a relatively small difference in BMI between the two groups where the averages of both groups fell in the overweight range and BMI had little association with the mitochondrial markers. Lastly, while physical activity is clearly associated with marked adaptations in mitochondrial number and function, this study sought to remove this element by including sedentary individuals and excluding individuals who were physically active. However, future studies are needed to evaluate the impact that physical activity may have on the mitochondrial capacity in older adults with ICF. Additional studies are also warranted to investigate whether the aforementioned mitochondrial mechanisms are a cause or consequence of ICF, whether these abnormalities are localized to skeletal muscle or systemic, and if there is any crosstalk between the musculoskeletal and nervous systems which would amplify the sensations of ICF.

## CONCLUSIONS

The aim of the present study was to investigate whether abnormalities in skeletal muscle mitochondria were associated with idiopathic chronic fatigue in older adults. More specifically, we hypothesized older adults with ICF exhibited 1) impairments in mechanisms regulating mitochondrial biogenesis signaling, 2) reductions in ETC complex expression, and/or 3) disruptions in the balance of oxidative damage and antioxidant capacity. Overall, our data points to impairments in mitochondrial number, biogenesis and electron transport chain activity as the primary perpetrators underlying the cellular mechanisms of ICF in older adults. Thus, interventions designed to target these mitochondrial impairments may be efficacious in combating fatigue and/or the perceived sensations of fatigue.

## EXPERIMENTAL METHODS

### Participants

A total of 61 sedentary men and women 65+ years old were enrolled in the study. The following exclusionary criteria were created to limit disease conditions that are overtly related to fatigue symptoms: depressive symptoms (CES-D≥16), untreated sleep apnea, alcohol or substance abuse, body mass index ≥ 35 kg/m^2^, degenerative neurological diseases, inflammatory disease, any pulmonary disease, cancer in past year, stroke in the past 6 months, fracture or joint replacement in past 6 months, insomnia being treated with hypnotic medication more than 2 nights/week, terminal illness, severe cardiac disease (NYHA Class III or IV congestive heart failure, clinically significant aortic stenosis, history of cardiac arrest, use of a cardiac defibrillator, or uncontrolled angina), severe sensory impairment, use of long-acting benzodiazepines or antipsychotic medication, living in a nursing home, actively participating in a formal exercise program within the past 3 months— defined as 20+ minutes of formal exercise at least once a week, hypothyroidism: T4 < 5 μg/dl or normal T4 and elevated TSH > 20 μU/ml, significant cognitive impairment defined as a known diagnosis of dementia or a Mini-Mental State Exam (MMSE) < 24, uncontrolled hypertension, anemia, acute infection or fever within 4 weeks, abnormal blood potassium, sodium or calcium levels, and hyperglycemia. Prior to enrollment in the study, all participants provided written informed consent using documents approved by the University of Florida Institutional Review Board.

### Functional assessment of chronic illness therapy (FACIT) fatigue scale

The FACIT Fatigue Scale (FACIT-F), a component of the FACIT Measurement System (http://www.facit.org/FACITOrg/Questionnaires) originally developed for patient reported outcomes in cancer and redeveloped for all chronic illnesses, was used to assess fatigue [67]. The scale assesses 13 self-reported items that relate to fatigue during daily activities over the past 7 days. It has been validated in a variety of disease conditions and in older adults [67-70]. Each item asked about a component of fatigue on a four point Likert scale (4 = not at all fatigued to 0 = very much fatigued) [71]. The total FACIT Fatigue Score was calculated according to a standard protocol and referenced to normative data [69, 72] (http://www.facit.org/FACITOrg/Questionnaires). ICF and NF groups were established as values that were 2 standard deviations of normative data [69] with FACIT fatigue scores that fell within ranges of ≤ 35 (ICF) and ≥ 42 (NF). To ensure that fatigue symptoms were not transient, the FACIT-F was administered on two separate occasions separated by greater than one week. The correlation between the two FACIT-F questionnaires that were administered more than one week apart from each other was 0.94 (*p* < 0.001) and a slope that followed the line of identity (Beta = 0.95). Participants who reported fatigue (or non-fatigue) symptoms outside the range were excluded for having transient fatigue that would not be consistent with idiopathic chronic fatigue.

### Muscle biopsies

Skeletal muscle samples were obtained under local anesthesia from the vastus lateralis using the Bergstrom muscle biopsy technique [73]. Samples were immediately weighed and remaining tissue frozen in liquid nitrogen and stored at -80 C until further analysis.

### Cytochrome c oxidase (COX) enzyme activity

Whole tissue was diluted to a 2-fold concentration in a buffer (0.1 M KH2PO4 + 2 mM EDTA, pH 7.2) and sonicated (3 x 5 s) on ice. Following a brief spin, the supernatant was removed and enzyme activity determined by the maximal oxidation rate of completely reduced cytochrome c, evaluated as a change in absorbance at 550 nm using a multi-detection microplate reader (Synergy HT, Biotek Instruments, Winooski, VT) [36, 37].

### Immunoblotting

Whole muscle tissue homogenates were prepared as previously described [36, 37]. Protein extracts from muscle homogenates were separated on 12-15% SDS-polyacrylamide gels and transferred to nitrocellulose membranes using an electroblotting transfer apparatus. Nitrocellulose membranes were blocked for 1 h with 5% skim milk in 1 × TBS-T (Tris-buffered saline Tween-20: 25 mM Tris-HCl, 1 mM NaCl, 0.1% Tween 20, pH 7.5). Membranes were incubated with primary antibodies overnight at 4 °C directed against phosphorylated-AMP-activated protein kinase-α (Thr172) (AMPKα; 1:500; Cell Signaling; #2531S), total-AMPKα (1:500; Cell Signaling, #2532), Sirt1 (1:500; Cell Signaling, #8469S), Sirt3 (1:100; Santa Cruz; sc-365175), PGC-1α (1:500; Calbiochem; #516557), NRF-1 (1:500; Rockland Immunochemicals; #200-4001-869), TFAM (1:500; Calbiochem; DR1071), cytochrome c (1:1500; Santa Cruz, sc-8385), Mfn-1 (1:1000; Sigma; M6319), Mfn-2 (1:1000; Sigma; M6444), Drp-1 (1:500; BioRad; 611112), Fis-1 (1:1000; Alexis Biochemicals; 210-907-R100) 4-HNE (1:500; Abcam; ab46545), MnSOD (1:2000; Santa Cruz; sc-30080), Catalase (1:2000; Abcam; ab16731), GPx (1:1000; Abcam; ab22604), and electron transport chain subunit proteins using a total OXPHOS antibody cocktail (1:1000; Mitosciences; #MS604). All commercially available antibodies have been extensively used in the literature. Ponceau S staining was used to normalize for the amount of protein loaded as previously described [24, 37]. Membranes were washed (3 × 5 min) with TBS-T then blocked with the appropriate secondary antibody at room temperature for 1 h, then washed again (3 × 5 min) with TBS-T and signals were detected using enhanced chemiluminescence (ECL; Santa Cruz). Films were imaged and analyzed using Silk Scientific “Un-Scan-Itgel” gel analysis software measuring pixel density while correcting for background intensity.

### Other measurements

Medical history and demographics (i.e. race, education, social status, mobility impairment, and self-rated health) were reported through clinic interview. Depressive symptoms were measured with the short form Center for Epidemiological Studies Depression Scale (CES-D) [74], and cognitive function was measured with the Mini Mental State Examination (MMSE) [75], Blood samples were used to ascertain sub-clinical conditions that may be responsible for fatigue symptoms (e.g. anemia). Blood was collected according to standard clinical protocols *via* venipuncture and assays were conducted in a Clinical Laboratory Improvement Amendments certified laboratory (CLIA certified).

### Statistical analysis

Differences between groups of ICF and NF subjects were analyzed by using one-way analysis of variance (ANOVA). Correlations between variables were investigated using Pearson product moment correlation analyses. Differences were considered statistically significant if *P* < 0.05 and *P*-values < 0.10 were also noted. Data are presented as means ± standard errors (SE) unless otherwise noted.
